# Using objective clinical metrics to understand the relationship between the electronic health record and physician well-being: observational pilot study

**DOI:** 10.1192/bjo.2021.993

**Published:** 2021-09-21

**Authors:** Matthew J. Mosquera, Heather Burrell Ward, Christopher Holland, Robert Boland, John Torous

**Affiliations:** Department of Psychiatry, Brigham and Women's Hospital, Harvard Medical School, USA; Department of Psychiatry, Brigham and Women's Hospital, Harvard Medical School, USA; Digital Health Department, Massachusetts General Hospital, USA; Department of Psychiatry, Brigham and Women's Hospital, Harvard Medical School, USA; Digital Psychiatry Division, Beth Israel Deaconess Medical Center, Harvard Medical School, USA

**Keywords:** Physician well-being, well-being, burnout, electronic health record, electronic medical record

## Abstract

**Background:**

Electronic health records (EHRs) are a significant contributor to physicians’ low satisfaction, reduced engagement and increased burnout. Yet the majority of evidence around EHR and physician harms is based on self-reported screen time, which may both over- and underreport actual exposure.

**Aims:**

The purpose of this study was to examine how objective EHR use correlates with physician well-being and to develop preliminary recommendations for well-being-based EHR interventions.

**Method:**

Prior to the onset of COVID-19, psychiatry residents and attending physicians working in an out-patient clinic at an academic medical centre provided consent for access to EHR-usage logs and completed a well-being assessment made up of three scales: the Maslach Burnout Inventory, the Urecht Work Engagement Scale and the Professional Quality of Life Measure. Survey responses and objective EHR data were analysed with descriptive statistics.

**Results:**

Responses were obtained from 20 psychiatry residents (total eligible residents *n* = 27; 74% participation) and 16 clinical faculty members (total eligible faculty *n* = 24; 67% participation) with an overall response rate of 71% (total eligible residents and faculty *n* = 51 and total residents and faculty who completed survey *n* = 36). Moderate correlations for multiple well-being domains emerged in analysis for all participants, especially around the time spent per note and patient visits closed the same day.

**Conclusions:**

EHR-usage logs represent an objective tool in the evaluation and enhancement of physician well-being. Results from our pilot study suggest that metrics for note writing efficiency and closing patient visits the same day are associated with physician well-being. These metrics will be important to study in ongoing efforts involving well-being-based EHR interventions.

## Background

Some degree of work-related stress in a physician's life is normal and unavoidable. However, when work-related stressors become overwhelming and unmanaged, there is an adverse impact on physician well-being. For instance, with increasing workplace stressors, burnout (a syndrome characterised by emotional exhaustion, low sense of personal accomplishment and depersonalisation^1^) may ensue, engagement (a positive, fulfilling, work-related state of mind that is characterised by vigour, dedication and absorption^2^) may suffer, and overall quality of life may plummet. An important note, when it comes to comprehensive assessment of physician well-being, is that it is essential to consider both positive and negative constructs; hence the inclusion of engagement and quality of life. That said, initial efforts in the field of physician well-being have focused on burnout, and how to combat it, justifiably so given the damage associated with it.

Burnout is associated with impaired clinical performance, suicidal ideation and increased frequency of medical errors. Nearly half of US physicians experience symptoms of burnout,^3^ which costs an estimated $4.6 billion to the US healthcare system.^4^ High prevalence rates for burnout have been observed across various medical specialties and at elevated levels throughout all years in residency and fellowship training.^3^ Within psychiatry, 78% of 2084 psychiatrists in a 2019 survey reported high burnout levels.^5^

Although there any many causes of physician burnout, one that has been the subject of increasing scrutiny is technology and especially electronic health record (EHR) use as a prominent source of professional dissatisfaction.^6^ Furthermore, in the COVID-19 virtual era where clinical care is increasingly provided via screens and EHR portals, understanding the impact of EHRs on burnout is more important than ever. Prior research on burnout and technology use offers increasing evidence around the relationship of burnout and EHR usage. A recent study found that excessive time (>90 min) spent on the EHR outside the workday and high clerical task burden (>60 min) increased physician risk for higher burnout, decreased work–life integration and lower professional satisfaction.^7^

Unpacking this relationship, a 2013 study conducted by the RAND health corporation and the American Medical Association found that inefficient data entry, poor usability of EHR products and lack of universal information exchange between EHR platforms had an impact on physician satisfaction levels.^6^ Similarly, within a 2012 nationwide survey of 1515 trainees across 24 specialties, 92% of residents reported that documentation obligations were excessive and, as a result, 90% felt time spent with patients has been compromised and 73% felt clinical documentation has had a negative effect on patient care.^8^ Finally, a 2018 pilot survey study of psychiatry residents and faculty demonstrated a strong positive correlation between self-reported EHR use and physician burnout.^9^

## Aims

The plethora of research demonstrating that physicians feel clinical documentation is excessive and detrimental to not only patient care but also to physician well-being is not particularly surprising. These important studies and data points, although valuable, all lack the detailed understanding of mechanisms of action required to develop specific solutions to enhance physician well-being. The more nuanced question, that has gone unanswered and remains critical for building solutions, is how exactly does interaction with the EHR have an impact on physician well-being. Answering this question requires objective data-driven exploration. Thus far, physician self-report usage of EHR has not been proven reliable compared with objective usage logs.^10^ And more importantly, no previous studies have utilised individual usage logs to explore how the EHR has an impact on physician well-being. Thus, we undertook this project to explore this important intersection (meaning the relationship between objective EHR usage logs and physician wellness) and gap in the existing literature. Based on trends in prior studies, our team's personalised experience as practicing clinicians using the EHR and the ability to craft subsequent interventions, we hypothesised that more burnout-related symptoms would be associated with greater time spent writing notes, increased time spent in chart review, increased length of notes, fewer patient visits closed the same day, and overall more time spent in the EHR system.

This study aimed to:

- explore how EHR use correlates with physician well-being (burnout, engagement, and quality of life) via retrospective analysis of individual EHR usage and well-being survey data;
- identify EHR-usage metrics that provide utility with regard to physician well-being; and
- develop preliminary recommendations for well-being-based EHR interventions and identify areas important for further investigation.

## Method

### Design, setting and participants

This study used a cross-sectional design to collect well-being survey data and back-end EHR-usage logs to gather data on use patterns for both attending and resident psychiatrists. It was conducted at an academic medical centre located in Boston, Massachusetts, where physicians used a comprehensive EHR that had been implemented roughly 5 years before initiation of this study. As a result of the functionality of the EHR-metrics collection tool, which tracks usage logs exclusively in ambulatory care settings, participants were limited to the out-patient clinic. With institutional review board approval, we distributed self-administered pencil-and-paper surveys to all residents and faculty staff within the out-patient psychiatric offices. Inclusion criteria for participants included residents and faculty attending physicians with either part-time or full-time out-patient clinic practice. Exclusion criteria included providers with out-patient clinic schedules meeting on a monthly or more infrequent basis. At the time of the survey distribution, according to out-patient administrative staff records, there were 24 and 27 eligible attending and resident psychiatrists, respectively.

### Data collection

We administered the same survey to residents and faculty staff in February 2020. Participants were recruited via work email to notify them of their eligibility for the study. All surveys were delivered to secure individual mailboxes and then self-administered, to be returned to lock-boxes in the psychiatry department. Following delivery of survey to their mailboxes, participants were allowed 2 weeks to complete the survey. No incentives were provided. All responses were anonymised and results were transcribed into a secure database. All analysis was performed in the R programming language and using the rcorr function,^11^ which computes a matrix of Pearson's *r* correlations and offers summary statistics. The *a priori* alpha level for correlations was set as *P* < 0.05.

The survey included three internationally recognised scales on well-being constructs (engagement, burnout and quality of life) for a multifaceted appraisal of physician well-being, as well as demographic questions, subjective questions on usage of the EHR, and subjective questions on sleep and exercise. A copy of the survey, excluding well-being scales, is available in the Supplementary Appendix available at https://doi.org/10.1192/bjo.2021.993.

For well-being scales, we included the Maslach Burnout Inventory (MBI), a validated measure for burnout, and specifically chose the human services version of the measure adapted for medical personnel.^1^ The MBI is comprised of three subscales: emotional exhaustion, depersonalisation and personal accomplishment.

To assess engagement, we selected the Utrecht Work Engagement Scale (UWES-15), which contains three, five-question subscales: vigour, dedication and absorption.^2^

Quality of life was assessed using the Professional Quality of Life (ProQOL) scale, a popular measure of compassion fatigue and compassion satisfaction for healthcare professionals, which includes three subscales: burnout, secondary traumatic stress and compassion satisfaction.^12^

For well-being survey responses, we selected six subgroup domains (three with positive directionality and three with negative directionality) to include in correlation analysis alongside EHR-user metric data. We elected to include the total score for the UWES, the compassion satisfaction and secondary traumatic stress subgroups for the ProQoL, and all three subdomains of the MBI (there is no total score subgroup available for the ProQoL or MBI). We did not include the burnout subgroup for the ProQoL given redundancy alongside the MBI and selected the total UWES score as opposed to the three individual subgroups in order to streamline results. well-being subgroup definitions used in correlation analyses are available in Supplementary Table 1.

For EHR-usage metrics, we utilised 3-month aggregated individual-user data, as opposed to monthly data given variation in the month-to-month schedules of residents. In order to provide broad exploration of user interaction with the EHR, we selected six usage metrics across categories of note writing (both time spent and length of notes), chart review, time in system and visits closed the same day given prior research suggested these may be related to burnout. The EHR metric descriptions used in correlation analyses are available in the Supplementary Table 2.

### Ethics statement

The authors assert that all procedures contributing to this work comply with the ethical standards of the relevant national and institutional committees on human experimentation and with the Helsinki Declaration of 1975, as revised in 2008. All procedures involving human patients were approved by Massachusetts General Brigham Institutional Review Board Protocol #:2019P002484. Informed consent for publication was obtained from all individual participants included in the study prior to administration of survey.

## Results

The overall response rate for all eligible participants was 71% (total eligible residents and faculty/attending physicians *n* = 51 and total residents and faculty/attending physicians who completed survey *n* = 36), and all submitted surveys were completed. Representing 20 of the residency's 27 eligible trainees with a 74% response rate, there were 6 second-year residents (PGY2s total eligible second-year residents *n* = 10), 10 third-year residents (PGY3s total eligible third-year residents *n* = 10) and 4 fourth-year residents (PGY4s; total eligible fourth-year residents *n* = 7) who completed the survey. Sixteen of the 24 clinical faculty staff who were offered the opportunity to take part in the survey completed it, a 67% response rate. Demographics for participants are provided in Table 1.

**Table 1:** Participant demographics by training/work experience

	Total sample (*n* = 36), *n* (%)	PGY2 (*n* = 6), *n* (%)	PGY3 (*n* = 10), *n* (%)	PGY4 (*n* = 4), *n* (%)	All residents (*n* = 20), *n* (%)	Junior attending^a^ (*n* = 8), *n* (%)	Senior attending (*n* = 8), *n* (%)	All attending physicians (*n* = 16), *n* (%)
Age, years								
26–35	24 (66.7)	6 (100)	10 (100)	4 (100)	20 (100)	4 (50)	n/a	4 (25)
36–45	8 (22.2)	n/a	n/a	n/a	n/a	4 (50)	4 (50)	8 (50)
46–55	1 (2.8)	n/a	n/a	n/a	n/a	n/a	1 (12.5)	1 (6.3)
56–65	2 (5.6)	n/a	n/a	n/a	n/a	n/a	2 (25)	2 (12.5)
66–75	1 (2.8)	n/a	n/a	n/a	n/a	n/a	1 (12.5)	1 (6.3)
Gender								
Female	27 (75)	5 (83.3)	7 (70)	3 (75)	15 (75)	6 (75)	6 (75)	12 (75)
Male	9 (25)	1 (16.7)	3 (30)	1 (25)	5 (25)	2 (25)	2 (25)	4 (25)

PGY, post graduate year (i.e. PGY2 corresponds to a second-year resident); n/a, not applicable.

a. Junior attending as defined by <5 years practice as an attending psychiatrist.

Although there was missing survey data for certain survey questions, data categories included in analysis, both for survey responses and EHR metrics, did not contain any missing data. All participants completed the MBI, ProQOL and UWES and mean scores for each scale and subdomains are reported in Table 2.

**Table 2:** Mean Maslach Burnout Inventory, Utrecht Work Engagement Scale, Professional Quality of Life Scale scores for subdomains

	All residents (*n* = 20), mean (s.d.)	All attending physicians (*n* = 16), mean (s.d.)	All participants (*n* = 36), mean (s.d.)
Maslach Burnout Inventory			
Depersonalisation	14.3 (7.6)	8.3 (6.4)	11.6 (7.8)
Emotional exhaustion	30.6 (12.1)	29.5 (10.2)	30.1 (11.5)
Personal accomplishment	32.8 (5.0)	33.7 (3.7)	33.2 (4.5)
Utrecht Work Engagement Scale			
Dedication mean score	19.3 (3.6)	22.9 (3.8)	20.9 (4.2)
Vigour mean score	18.9 (5.9)	22.6 (4.8)	20.5 (5.8)
Absorption	19.0 (5.2)	22.7 (5.6)	20.6 (5.8)
Total engagement	57.1 (13.0)	68.2 (12.3)	62.0 (14.1)
Professional Quality of Life Scale			
Compassion satisfaction	33.0 (6.6)	37.0 (5.2)	34.8 (6.5)
Secondary trauma stress	23.0 (6.5)	23.4 (5.4)	23.2 (6.2)
Burnout	26.7 (7.3)	25.5 (3.9)	26.1 (6.1)

When comparing residents (*n* = 20) and attending physicians (*n* = 16) notable differences were that the residents were significantly less engaged (*P* = 0.016) and experienced increased depersonalisation (*P* = 0.020) compared with attending physicians. All other well-being subgroups did not produce significant differences.

**Fig. 1:**
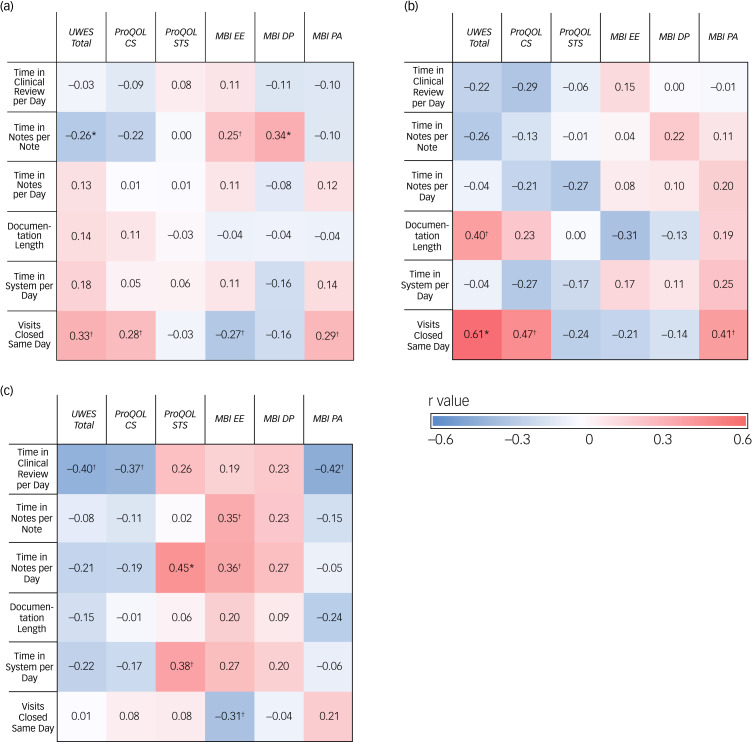
Correlation matrices for electronic health record (EHR) metrics and well-being survey responses. (a) All participants, *n* = 36; (b) attending physicians *n* = 16; and (c) residents, *n* = 20. CS, compassion satisfaction; DP, depersonalisation; EE, emotional exhaustion; MBI, Maslach Burnout Inventory; PA, personal accomplishment; ProQOL, Professional Quality of Life Scale; STS, secondary traumatic stress; UWES, Utrecht Work Engagement Scale. ^†^*P* < 0.2, *P < 0.05.

Correlation analysis is depicted in Fig. 1. Results are presented in the form of Pearson's correlation coefficient (*r*) with higher *r*-values indicating stronger correlation and thus a higher likelihood of significant finding (i.e. lower *P*-values). Correlations between additional EHR metrics and well-being survey responses are available on request from the authors.

Among all participants (*n* = 36), multiple well-being domains were moderately correlated with note writing time and number of visits closed on the same day. Specifically, more time spent writing each note was associated with significantly increased levels of depersonalisation (*r* = 0.34, *P* = 0.045), as well as a reduction in overall engagement (*r* = −0.26, *P* = 0.12) and higher levels of emotional exhaustion (*r* = 0.25, *P* = 0.15).

Furthermore, participants that closed more patient visits the same day reported less emotional exhaustion (*r* = −0.27, *P* = 0.11), higher levels of engagement (*r* = 0.33, *P* = 0.052), compassion satisfaction (*r* = 0.28, *P* = 0.10) and sense of personal accomplishment (*r* = 0.29, *P* = 0.082). User metrics for time spent on a per day basis in notes, chart reviews or the EHR system did not generate strong correlations between well-being survey results.

Mean 3-month usage metric data is provided in Table 3. Regarding how perceptions of EHR use compared with objective metrics, participants overestimated both time in the system per day by 4.76 h and time outside schedule hours by an average of 45.7 min. (Note: time outside scheduled hours meaning >30 min before first appointment, and/or >30 min after last appointment. Objective data for this metric were unavailable for PGY2s because of limited clinic time.)

**Table 3:** Mean electronic health record (EHR) usage metrics for 3-month data

EHR metric	All residents (*n* = 20), mean (s.d.)	All attending physicians (*n* = 16), mean (s.d.)	All participants (*n* = 36), mean (s.d.)
Time in clinical review per day	7.5 (3.8)	14.0 (7.9)	10.7 (6.8)
Time in notes per note	20.4 (10.6)	13.0 (7.7)	17.1 (10.1)
Time in notes per day	23.7 (10.1)	46.7 (27.8)	33.9 (23.1)
Documentation length^a^	8043.5 (2681.8)	8453.4 (3659.6)	8225.7 (3160.6)
Time in system per day	49.6 (17.4)	99.8 (40.3)	71.9 (38.9)
% Visits closed same day	0.5 (0.3)	0.7 (0.3)	0.6 (0.3)

a. Average number of characters per note written by the participant during the reporting period

As far as differences between groups are concerned, there was one significantly different result; residents spent more time in notes per note compared with the time spent on this by attending physicians (*P* = 0.029). After taking into account that attending physicians spend nearly double the amount of time in the system per day compared with residents, there were no other significant differences in EHR metrics between groups.

Beginning with the results for the resident participants, the time in notes per day metric produced moderate correlations with secondary traumatic stress (*r* = 0.45, *P* = 0.045) and emotional exhaustion (*r* = 0.36, *P* = 0.12). The times in notes per note metric also generated a strong correlation with the emotional exhaustion (*r* = 0.35, *P* = 0.13) domain. All correlations between the documentation length and well-being subgroups failed to produce strong results.

For attending physicians, the note writing metrics (both per note and per day) did not produce strong correlations with well-being data. Unlike for the residents, there was a strong positive correlation for the attending group between documentation length and engagement (*r* = 0.4, *P* = 0.12). For attending physicians, total time in the system per day did not significantly correlate with any well-being subgroups.

## Discussion

Results from our study suggest noteworthy correlations between multiple EHR-usage metrics and unique components of physician well-being. Although our results varied between the two participant groups analysed, broad trends included that more time spent writing each note is associated with staff who are emotionally exhausted, less engaged, and, most of all, experiencing higher levels of depersonalisation while at work. Overall levels of burnout, as measured by the MBI, were similar to those previously measured in a comparable study population.^9^ As far as physician perceptions are concerned, overestimations in both total time spent in system and time outside scheduled hours were comparable with previous studies.^10,13^

### Implications

Our results have immediate clinical relevance. Residents and attending physicians may benefit from well-being initiatives that either enable and/or incentivise staff to close more patient visits the same day and spend less time writing each note. For residents specifically, we would advise interventions geared towards enhancing EHR-user efficiency with a focus primarily on note writing and also chart review, both of which would likely have an impact on the total time spent in the system. These efficiency-focused interventions could also be applied to attending physician; however, for attending physicians it appears the strongest emphasis should be placed on empowering staff to close patient visits the same day. To move beyond these preliminary recommendations, it will be necessary for further research to include before and after sampling for EHR well-being-based interventions, as well as including a large number of participants to provide more cohort-specific analysis. We acknowledge that any proposal should be tailored to the level of training and/or practice and that these results may not generalise.

In terms of studying burnout, both our methods and results also offer actionable information. Objective EHR metrics represent a valuable tool that is superior to subjective reports in their ability to eliminate recall bias. This is especially true for the more nuanced ways physicians interact with the EHR, such as time spent in chart review or time spent in notes per note that cannot be reliably estimated via subjective polling. Regarding the selection of metrics to study, it is therefore critical to first determine the goal of an analysis while also having in mind the target population and the capability for future interventions. Prior to gathering data for our study, based on previous studies^9^ and clinical experience, we hypothesised that certain EHR-usage metrics would correlate with physician well-being. These metrics of focus were also selected based on their modifiable nature and potential for use in future interventions.

### Limitations

When interpreting the results of this pilot study, several limitations must be noted. Regarding analysis, we recognise that reporting on associations between variables does not imply causal relationships. It is thus unclear if characteristics of enhanced physician well-being such as high engagement level leads to more visits closed each day, or vice versa. Furthermore, external validity is restricted because this study was conducted at a single out-patient practice with EHR- user metrics confined to the ambulatory care setting. Moreover, although EHR-usage metrics provide objective and detailed measures for user practices, they lack clinical workflow context and therefore do not capture the quality of individual-user experiences with the EHR. For instance, the usage metric for closing patient visits the same day appears to have a broad association across multiple well-being domains. The lack of significant findings for this EHR metric may be secondary to a characteristic of the teaching clinic as residents frequently rely on supervising attending physicians to enter a co-signature before visits can be closed.

Additionally, because of the small sample size of this pilot study, breakdown into training cohorts was restricted to two groups (all residents and all attending physicians) while still maintaining the ability to demonstrate either significant results or results with trends towards significance. The differences between the two participant groups underscore the valuable correlations provided by performing analysis at the level of training cohorts. This variability, which may be attributed to multiple factors including difference in out-patient case-loads and work schedules, suggests that there is not a ubiquitous relationship with the EHR for physicians even within one specialty. Accordingly for future studies, the more cohort-specific analyses the better in order to enhance external validity.

Despite these limitations, this study is novel in its identification of which EHR-usage metrics demonstrate utility in assessment of physician well-being. This information serves as proof of concept for inclusion of EHR-user metrics in all future well-being-based EHR interventions. Furthermore, the strong correlations identified in this study involving metrics for closing patient visits the same day and time spent writing notes can serve as recommendations for the aforementioned well-being-based EHR interventions. The methodology of this study may also serve as a framework for larger-scale studies involving larger samples, different specialties, several institutions and/or multiple times points to further characterise the relationship between EHR use and physician well-being. In addition, further research may also usefully include exploration of the impact of COVID-19 and transition to telehealth on EHR-user practices and physician well-being.

## Data Availability

The data-sets used and/or analysed during the current study are available from the corresponding author on reasonable request.

## Supplementary material

For supplementary material accompanying this paper visit http://dx.doi.org/10.1192/bjo.2021.993. click here to view supplementary material
