# Supplementary material for: Using objective clinical metrics to understand the relationship between the electronic health record and physician well-being: observational pilot study
Source: BJPsych Open. 2021 Sep 21;7(5):e174. doi: 10.1192/bjo.2021.993 (PMC8485348; doi:10.1192/bjo.2021.993)
Supplement: Supplementary file 1 [file bjosup.zip › S2056472421009935sup001.docx]

Background Questions for Wellness Study:

**Gender:** *(Please circle)*

F M

**Age:** *(Please circle)*

26-35 36-45 46-55 56-65 66-75 76-85

**Position:** *(Please circle)*

Resident PGY: 2 / 3 / 4

or

Faculty (years in practice): <5 years 5-10 years 11-15 years ≥16 years

Faculty Please Average number of scheduled **hours per week** **for direct patient**

Answer this 🡪 **care in the outpatient setting at** ***BLINDED*** (*ie. Do not include private*

*practice, resident education, time on consults, admin time or inpatient time*)?

*-This question meant to*

*stratify which faculty members*

*spend more time in outpatient care ______________________________*

*for analysis purposes*

*(Please provide best estimates for your* ***BLINDED***  *use on a* ***typical day*** *in the* ***OUTPATIENT SETTING****)*

**Imagine a typical day in the outpatient office….**

- How many TOTAL hours do you spend on ***BLINDED*** during that day?_____________

- How many hours do you spend WRITING NOTES on ***BLINDED*** during that day? ________________

- How many hours do you spend REVIEWING CHARTS on ***BLINDED*** during that day? ______________

-How many hours do you spend on ***BLINDED*** **outside of your scheduled hours** (meaning >30min before first appointment, and/or >30min after last appointment) working on patient encounters **on that day**? _________________

-What percentage of outpatient encounters closed

(ie. note completed) **on same day**? ______________

-How many hours do you spend on ***BLINDED*** **on days with no scheduled** patients (ie. Weekends or administrative days) wrapping up patient encounters **from previous days**? _______________

Do you feel that the **outpatient notes** you are required to write are:

too short (1) ----too long (10)

(*Please circle)*

1 2 3 4 5 6 7 8 9 10

*(Please provide best estimates)*

Hours of exercise **per day** on average? ____________

Hours of sleep **per night** on average? ______________

- - - - - - - - - - - - - - - - - - - - - - - - - - - - - - - - - - - - - - - - - - - - - - - - - - - - - - - - - - - - - - - - - - - - - - - -

***BLINDED*** Net ID: __________________

(*aka what you enter to log into* ***BLINDED****computers under username*)

**NOTE: This piece of information will be discarded after linking process is complete for clinical metric data**
