# Supplementary material for: Using objective clinical metrics to understand the relationship between the electronic health record and physician well-being: observational pilot study
Source: BJPsych Open. 2021 Sep 21;7(5):e174. doi: 10.1192/bjo.2021.993 (PMC8485348; doi:10.1192/bjo.2021.993)
Supplement: Supplementary file 1 [file bjosup.zip › S2056472421009935sup002.docx]

**Survey Questions**

Section 1: Background Questions

**Gender:**

- **M**
- **F**

**Age:**

- 26-35
- 36-45
- 46-55
- 56-65
- 66-75
- 76-85

**Position:**

Resident PGY:

- 2
- 3
- 4

or

Faculty (years in practice):

- <5 years
- 5-10 years
- 11-15 years
- ≥16 years

Faculty Please Answer this:

- Average number of scheduled hours per week for direct patient care in the outpatient setting (*ie. Do not include private practice, resident education, time on consults, admin time or inpatient time*)? ________

Section 2: EHR Use

*(Please provide best estimates for your EHR use on a typical day in the OUTPATIENT SETTING)*

Imagine a typical day in the outpatient office….

- How many TOTAL hours do you spend on EPIC during that day?
- How many hours do you spend WRITING NOTES on EPIC during that day?
- How many hours do you spend REVIEWING CHARTS on EPIC during that day?
- How many hours do you spend on EPIC outside of your scheduled hours (meaning >30min before first appointment, and/or >30min after last appointment) working on patient encounters on that day?
- What percentage of outpatient encounters closed (ie. note completed) on same day?
- How many hours do you spend on EPIC on days with no scheduled patients (ie. Weekends or administrative days) wrapping up patient encounters from previous days?
- Do you feel that the outpatient notes you are required to write are:

too short (1) ----too long (10)

(*Please circle)*

1 2 3 4 5 6 7 8 9 10

Section 3: Exercise and Sleep

- Hours of exercise per day on average?
- Hours of sleep per night on average?
