# Supplementary material for: Using objective clinical metrics to understand the relationship between the electronic health record and physician well-being: observational pilot study
Source: BJPsych Open. 2021 Sep 21;7(5):e174. doi: 10.1192/bjo.2021.993 (PMC8485348; doi:10.1192/bjo.2021.993)
Supplement: Supplementary file 1 [file bjosup.zip › S2056472421009935sup003.docx]

**Supplementary Table 2:** Metric descriptions

| **Metric** | **Definition** | **Denominator** |
| --- | --- | --- |
| Time in Clinical Review Per Day | Average amount of time each provider spent per day in clinical review activities, such as Chart Review | Total number of days that the provider logged in and worked during the reporting period |
| Time in Notes Per Day | Average amount of time each provider spent per day writing notes | Total number of days that the provider logged in and worked during the reporting period |
| Time in Notes Per Note | Average time spent writing a note for each note written per provider | Number of notes written during the reporting period |
| Documentation Length | Average number of characters per note written by the provider during the reporting period | Number of notes written during the reporting period |
| Time in System per day | Average number of minutes the provider was logged into the system per day | Total number of days that the provider logged in and worked during the reporting period |
| Visits Closed Same Day | The percentage of visits for the reporting period that were closed same day | Total visits closed during the week or unclosed from the prior week |
| 3-Month Reporting period as 10/27/2019-1/25/2020 | | |
