# Supplementary material for: Using objective clinical metrics to understand the relationship between the electronic health record and physician well-being: observational pilot study
Source: BJPsych Open. 2021 Sep 21;7(5):e174. doi: 10.1192/bjo.2021.993 (PMC8485348; doi:10.1192/bjo.2021.993)
Supplement: Supplementary file 1 [file bjosup.zip › S2056472421009935sup004.docx]

**Supplementary Table 1:** Wellness Subgroup Definitions

| **Wellness Survey** | **Subgroup** | **Definition** |
| --- | --- | --- |
| Utrecht Work Engagement Scale (UWES) | Total Score | A positive, fulfilling, work-related state of mind that is characterized by vigor, dedication, and absorption (2) |
| Professional Quality of Life Scale (Pro QoL) | Compassion Satisfaction  (CS) | The pleasure you derive from being able to do your work well (13) |
| Professional Quality of Life Scale (Pro QoL) | Secondary Traumatic Stress (STS) | A negative feeling driven by fear and work-related trauma (13) |
| Maslach Burnout Inventory (MBI) | Emotional Exhaustion  (EE) | Negative feelings of being emotionally overextended and exhausted by one’s work (1) |
| Maslach Burnout Inventory (MBI) | Depersonalization  (DP) | A negative unfeeling and impersonal response toward recipients of one’s service, care treatment, or instruction (1) |
| Maslach Burnout Inventory (MBI) | Personal Accomplishment (PA) | Positive feelings of competence and successful achievement in one's work with people (1) |
